# Targeting cancer associated fibroblasts to enhance immunotherapy: emerging strategies and future perspectives

**DOI:** 10.18632/oncotarget.27936

**Published:** 2021-07-06

**Authors:** Christopher J. Hanley, Gareth J. Thomas

**Affiliations:** ^1^School of Cancer Sciences, University of Southampton, Southampton, UK; ^2^Cancer Research UK and NIHR Southampton Experimental Cancer Medicine Centre, Southampton, UK

**Keywords:** tumour microenvironment, cancer associated fibroblasts, immunotherapy

## Abstract

Cancer associated fibroblasts are a prominent component of the tumour microenvironment in most solid cancers. This heterogeneous population of cells are known to play an important role in tumour progression and recent studies have demonstrated that CAFs may confer resistance to checkpoint immunotherapy, suggesting that targeting these cells could improve response rates. However, effective clinical strategies for CAF targeting have yet to be identified. In this editorial, we highlight current limitations in our understanding of CAF heterogeneity, and discuss the potential and possible approaches for CAF-directed therapy.

## INTRODUCTION

Immunotherapies are increasingly used as first-line treatments for solid cancers. However, only a subset of patients respond and the size of this subset varies significantly between tumour types. Researchers are investigating a multitude of strategies to improve these response rates and to develop novel methods of stimulating an anti-cancer immune response. Cancer associated fibroblast (CAF) targeting is one strategy for enhancing immunotherapy efficacy in pre-clinical models and may improve patient response rates. In a recent study [1], we showed that CAFs prevent CD8+ T-cells from infiltrating solid tumours by inducing CTLA4 upregulation, which led to abrogated immunotherapy (anti-PD1 and anti-cancer vaccination) efficacy. We also showed that NOX4 inhibition could reverse TGF-β1 mediated CAF activation, leading to reduced extra-cellular matrix (ECM) deposition, increased CD8+ T-cell infiltration into tumours and improved immunotherapy efficacy. In this editorial we discuss these findings and those from recent studies, with the aim of highlighting key areas for further investigation in order to advance translational research into targeting CAFs as an immunotherapy adjunct. We will focus on the what, when and how of CAF targeting: *what* fibroblast phenotypes or subpopulations could be targeted; *when* might CAF targeting be a useful addition to immunotherapy treatment regimens; and *how* CAFs could be targeted. For a more comprehensive description of and introduction to CAF biology, there are multiple excellent review articles that have been published recently [2–5].

### What are CAFs?

Since CAFs were first shown to have a role in tumour progression, researchers have tried in vain to identify a specific marker for these cells. The difficulty associated with this endeavour is likely two-fold. First, fibroblast markers are also poorly defined in normal physiology and these cells are commonly classified by the absence of lineage-restricted markers and cell morphology, although recent studies have identified fibroblast-specific gene expression profiles through single-cell (sc)RNA-sequencing [6]. Second, fibroblasts have been shown to exhibit significant heterogeneity within specific tissues and across different anatomical locales [7]. In the absence of well-defined and consistently used markers it is difficult to reconcile the variation observed between studies, where CAF phenotypes have been examined across different model systems or tumour types. However, many studies have shown that these cells frequently display an ‘activated’ phenotype in comparison to their control tissue counterparts and this activation most commonly manifests functionally in increased contractility, ECM deposition and/or inflammation associated gene expression [8–11]. CAF’s role in tissue contraction and ECM deposition has led to a long-standing association between these cells and myofibroblasts, which are also found during wound healing and fibrosis. As a result, α-smooth muscle actin (SMA) is widely used as a marker for CAFs, denoting a myofibroblast-like phenotype. SMA has also been used to demonstrate correlations between CAFs and poor survival rates in many solid tumours [12–15]. However, recent pancreatic cancer studies have generated controversy over whether the SMA+ CAF population found in many solid tumours is a tumour-promoting or tumour-suppressive entity [16, 17].

Recent technological advances, such as scRNA-sequencing, have provided an opportunity to evaluate CAF heterogeneity in an unsupervised manner: mitigating the need for cell sorting to generate cell-type specific gene expression profiles. This has led to numerous recent studies documenting fibroblast heterogeneity in human and murine tumours [18–24]. The most consistently observed finding from this research confirms the existence of myofibroblastic and inflammatory CAFs (myCAF and iCAF respectively). This research has also highlighted issues associated with using *ACTA2* (the gene encoding SMA) expression as the sole criteria for identifying CAFs or even myCAF, as *ACTA2+* smooth muscle cells and pericytes are also commonly found in the tumour microenvironment and these cells exhibit higher *ACTA2* expression levels than CAFs [6].

This heterogeneity prompts the question of which phenotypes should be targeted therapeutically. It remains unclear how different fibroblast subpopulations are involved in tumour progression and, of particular relevance here, in anti-tumour immunity. MyCAF gene signatures are upregulated in patients that failed to respond to immunotherapy [19, 20, 25, 26]. We showed that co-injection of myCAF and tumour cells causes CD8 T-cells to accumulate at the tumour periphery in a mechanism that is, at least in part, regulated by CTLA4 upregulation on the CD8 T-cells [1]. This exclusion effect has also been shown in human tumour cohorts [27] and other model systems [26]. Kieffer *et al*. showed that myCAF induce PD1 and CTLA4 expression on Tregs (CD4+ CD25+ FOXP3+ T-cells) in a positive feedback loop [20]. This is potentially critical to their role in response to anti-PD1 as PD1+ Tregs can lead to rapid tumour growth following treatment, due to amplification of their immunosuppressive properties [28].

The iCAF gene expression profile is characterised by up-regulation of many different cytokines. For example, IL6 and CCL2 which are known to regulate myeloid cell recruitment [29] as well as tumour progression [30]. These cells have also been shown to represent the principal tumour-promoting CAF subpopulation in pancreatic cancer model systems [11, 17]. However, Kieffer *et al*. showed that iCAF gene signatures were not linked to immunotherapy response in a human lung cancer cohort [20] and the precise role that this CAF subset plays in immunotherapy resistance remains unclear.

### When is CAF targeting likely to improve immunotherapy efficacy?

As described above, there is significant evidence to support myCAF targeting as a mechanism to enhance immunotherapies. However, in order to effectively translate these pre-clincal findings careful consideration for when this is most likely to achieve patient benefit is required. To date, CAF targeting strategies have not proved effective in the clinic [31–34] and there are currently no effective biomarkers for determining whether a patient is likely to benefit from such treatments. Therefore, no selection criteria for CAF abundance or phenotype are in place when recruiting patients to ongoing clinical trials. It is imperative that various CAF biomarkers are tested in ongoing clinical trials to enable continued improvement in the design of these studies.

It is tempting to speculate that any tumour with a high degree of myCAF involvement could benefit from CAF targeting. However, it is not clear whether these cells function similarly across cancer types. For example, in pancreatic cancer (the archetypal myCAF rich tumour) studies have consistently shown that at least a subset of myCAF are responsible for restraining tumour progression. Given these findings and the fact that pancreatic cancers are typically “immune cold” tumours, it is perhaps unlikely that myCAF targeting will be sufficient to improve immune checkpoint blockade in this setting.

Mariathasan *et al*. have provided the most clinically relevant evaluation of this conundrum to date, using RNA-sequencing data from an anti-PD-L1 trial in metastatic bladder cancer, to demonstrate that myCAF impact treatment efficacy. This study showed that a myCAF gene signature only had significant bearing on treatment response in patients with immune excluded tumours [26], highlighting myCAF-rich excluded tumours as a key cohort for testing CAF targeting strategies. We developed myCAF-rich models to study this group of patients [1], demonstrating potential for the use of NOX4 inhibition as a CAF targeting strategy in this setting and multiple studies have described similar responses to alternative myCAF targeting strategies [26, 35, 36].

The limitation of using descriptive analysis of human patient cohorts to identify those likely to benefit from CAF targeting is that you cannot account for precisely how targeting CAFs will modify the tumour microenvironment. A recent study by Tauriello *et al.* suggests that the potential for targeting myCAF could extend further than these immune excluded cases. In a model system that re-capitulates human microsatellite stable colorectal cancer, which has low tumour mutational burden and limited T-cell recruitment, TGF-β inhibition reduced myCAF abundance and promoted a T_H_1 adaptive immune response, resulting in increased CD8 T-cell activation and tumour clearance [37]. Furthermore, this study showed TGF-β inhibition combined with anti-PD-L1 treatment was effective in curing established metastases [37]. A notable difference between the model system used in this study and those frequently used to investigate CAF’s role in immunotherapy, is that this system elicits a spontaneous desmoplastic stromal response similar to that seen in human tumours. In our study [1], we found that tumour models which are commonly used to test immunotherapies (TC1, MC38, 4T1) contain very few myCAF. Therefore, we co-injected myCAF with tumour cells to investigate their role in anti-tumour immunity. In contrast to the Tauriello *et al.* model, we found that CAF targeting slowed tumour progression, but did not eliminate the tumour entirely. This could be due to CAF-independent immune evasion mechanisms; the tumour cells we used in these co-injection models grow rapidly without co-injecting myCAF, and it is likely they have developed intrinsic mechanisms for evading immune recognition. This also occurs in human tumours, as demonstrated by a recent study showing that malignant cells in immune infiltrated regions of human lung cancers, undergo immunoediting to restrict presentation of neoantigens [38]. It is possible that tumour cells in an “immune cold” myCAF-rich microenvironment, have not been subjected to the selection pressures that generate such immune evasion mechanisms, making them particularly vulnerable to anti-tumour immunity once the protective microenvironment is removed. These findings suggest that myCAF targeting may also present an exciting option for “heating-up” immune cold tumours.

### How can CAF be targeted to improve immunotherapy efficacy?

There are many strategies under investigation for targeting CAFs including depletion, blocking their tumour-promoting functions, inhibiting their activation, and skewing them to a normal or even tumour-suppressive phenotype.

The lack of specific markers for CAFs presents a significant barrier to depletion strategies. Despite this, CAF depletion was the first strategy to be shown to augment immunotherapy treatments. In a landmark study, Kraman *et al.* demonstrated that depleting Fibroblast Activation Protein (FAP)+ CAFs improved anti-cancer vaccination efficacy [39]. Following this study FAP+ CAFs have been extensively investigated and many pre-clinical studies have demonstrated the potential for depleting these cells in cancer treatment [40]. However, FAP expression is not limited to immunosuppressive stromal cells in the tumour microenvironment [41] and is also expressed on skeletal muscle and bone marrow stromal cells [42, 43]. This lack of specificity has highlighted the need for caution when targeting FAP+ stromal cells, as systemic depletion was shown to cause cachexia and anaemia [42, 43].

In the absence of specific methods to deplete immune-suppressive or tumour-promoting CAFs researchers have sought to identify the mechanisms of CAF-mediated immune suppression and then target these functions directly. The best described mechanism of CAF-mediated immunosuppression was discovered by further analysis of FAP+ CAF, which identified CXCL12/SDF-1 expression as critical to their immunosuppressive phenotype [44]. CAF mediated CXCL12 expression is now well described to play an important role in suppressing anti-tumour immunity, this was recently identified as a defining feature of the “CAF-S1” immunosuppressive subset of breast cancer myCAF [22] and blocking the interaction between CXCL12 and its cognate receptor CXCR4, with a clinically approved inhibitor (AMD3100), has shown efficacy in pre-clinical models [44, 45]. In our study [1], we showed that CTLA-4 blocking antibodies can also be used to overcome CAF-mediated exclusion of CD8+ T-cells in tumour models. Multiple studies have also shown that the ECM deposited by myCAF could be responsible for creating a physical barrier that CD8 T-cells are unable to cross. Proof of this principle was shown using a tumour slice model, which found that degrading peri-tumoural collagen using collagenase could enable CD8 T-cells to access tumour islands [46]. Building on this principle, inhibitors and enzymes that modify the ECM are undergoing testing as potential cancer treatments. These strategies include lysyl oxidase inhibitors [47] and hyaluronidase treatment [48]. However, early clinical trials investigating the combination of hyaluronidase and chemotherapy treatment in pancreatic cancer caused a significant reduction in overall survival rates compared to the control arm due to increased toxicity [49]. Furthermore, a recent study using genetically engineered pancreatic cancer models has called into question whether collagenous ECM plays an active role in suppressing T-cell recruitment to tumours. This study demonstrated that Col1a1 deletion in myCAFs increased immune suppression through increased recruitment of myeloid derived suppressor cells and reducing both CD3 T-cells and CD19 B-cells [50].

An alternative strategy to targeting a specific function of CAFs is to ‘normalise’ the phenotype of these cells. This is the strategy that we pursued in our study [1], using NOX4 inhibition to prevent and reverse myofibroblast activation. We have shown that NOX4 is an important regulator of TGF-β1 mediated myCAF activation [13] and that NOX4 inhibition can reduce ECM deposition and CAF-mediated tumour cell invasion and migration [1, 13, 51]. These data demonstrate the potential to target many of the tumour-promoting attributes associated with myCAF simultaneously. Alternative approaches to inhibit myCAF activation include TGF-β blockade [26, 36, 37] and angiotensin receptor inhibition [35]. The involvement of these pathways in myCAF activation has been appreciated for many years. However, their pleiotropic nature has mitigated their potential as therapeutic targets. Novel formulations of these inhibitors and/or increased specificity of delivery have demonstrated pre-clinical efficacy and clinical trials are underway to test whether this could translate into patient benefit (NCT02937272, NCT03563248). However, the recent discontinuation of a phase III trial evaluating a bispecific antibody targeting TGF-β and PD-L1 (bintrafusp alfa [M7824]; NCT03631706), as first-line treatment for advanced stage lung cancer patients with high PD-L1, shows there is still significant work to be done in order to effectively target these pathways in humans.

In contrast to inhibiting the pathways that regulate CAF activation, an emerging concept for CAF targeting is to stimulate pathways active in normal fibroblasts or stellate cells. The best described mechanism for this is the use Vitamin D/A receptor agonists in pancreatic cancer, which have been shown to reprogram CAFs to a quiescent state and enhance chemotherapy efficacy [52, 53]. It remains to be seen whether this treatment strategy could be effective in alleviating CAF-mediated immunosuppression, and it has been shown that vitamin D analogues can also significantly reduce T-cell effector function [54], which could impair the efficacy of these agents as immunotherapy adjuncts.

The success of both myCAF inhibition and quiescence stimulation strategies are contingent on the plasticity of fibroblast phenotypes. For many years it was thought that reverting myofibroblasts to a quiescent state was unlikely to be possible because the majority of these cells undergo apoptosis rather than reversion upon resolution of a wound healing response [55, 56]. However, recent research has shown that myofibroblast activation does not result in a state of permanent terminal differentiation. We showed that NOX4 inhibition is sufficient to not only prevent myCAF activation but also revert these cells to a more quiescent state. In contrast to TGF-β receptor inhibition, which was highly effective at preventing TGF-β mediated activation but not capable of reverting an established myofibroblast phenotype [1, 13]. This suggests that targeting downstream mediators of TGF-β signalling may provide greater efficacy when treating tumours with an established CAF-rich stroma. A key element in pursuing these phenotype-modulating therapeutic strategies, will be to carefully examine the resulting CAF phenotype in the tumour microenvironment. A recent study by Grauel *et al*. elegantly demonstrated the importance of this, showing that TGF-β blockade in murine cancer models not only led to reduced myCAF accumulation but also the emergence of an “interferon-licensed” fibroblast subpopulation [36]. These cells upregulated CXCL9/10, cytokines involved in the recruitment of T-cells and may be positively involved in regulating anti-tumour immunity and response to immunotherapy.

## CONCLUSIONS

In summary, there is significant pre-clinical data to suggest that CAF targeting could increase immunotherapy efficacy. However, it remains unclear what facets of the heterogeneous CAF phenotype are most important to their role in immune evasion in human tumours. It is likely that as research into CAF heterogeneity progresses to functional characterisation of the distinct subpopulations recently identified, we will develop a clearer understanding of how these cells contribute to disease progression. Further investigation is also required to determine when CAF targeting is likely to have a significant impact on immunotherapy efficacy. To address this, it will be essential to critically evaluate the strengths and weaknesses of different pre-clinical model systems in recapitulating the process of CAF activation and immune evasion found in human tumours. It is also important that we are able to learn from the many ongoing clinical trials by establishing reliable and consistently used biomarkers for important CAF phenotypes, such as iCAF and myCAF abundance.
